# Cost of Coexisting with a Relict Large Carnivore Population: Impact of Apennine Brown Bears, 2005–2015

**DOI:** 10.3390/ani11051453

**Published:** 2021-05-19

**Authors:** Andrea Galluzzi, Valerio Donfrancesco, Gianluca Mastrantonio, Cinzia Sulli, Paolo Ciucci

**Affiliations:** 1 Department of Biology and Biotechnologies, University of Rome La Sapienza, 00185 Rome, Italy; andrea.galluzzi@hotmail.com; 2 Department of Geography, University of Cambridge, Cambridge CB2 3EN, UK; vd308@cam.ac.uk; 3 Department of Mathematics (DISMA), Politecnico di Torino, 10129 Torino, Italy; gianluca.mastrantonio@polito.it; 4 Scientific Service, Abruzzo Lazio and Molise National Park, 67032 Pescasseroli, Italy; cinzia.sulli@parcoabruzzo.it

**Keywords:** human-carnivore coexistence, compensation costs, Human-bear conflict, human-dominated landscape, Italy, large carnivores, *Ursus arctos*

## Abstract

**Simple Summary:**

Efficiently addressing human-large carnivore conflicts is a conservation issue of increasing relevance, especially in human-dominated landscapes where impact on rural economies generates negative attitudes towards large carnivores and their conservation. We quantified patterns of bear impact on farms and the costs of compensation from 2005 to 2015 in the Abruzzo Lazio and Molise National Park (central Italy), an historical stronghold of the relict and highly imperiled Apennine brown bear population, where the park authority has been adopting conflict management approaches since the 1960s. Although the compensation program is rather costly (1490 ± 589 €/bear/year), the park policy has been increasingly integrated with prevention incentives, managing to effectively avert further increases in bear damages during the study period. Concurrently, local residents generally share a positive attitude towards bears, and the number of illegally killed bears decreased in the last decade. Despite this, our findings indicate there is still room for improvement in local conflict management, and that a more efficient use of conservation funds would benefit from increased monitoring, integrated prevention, conditional compensation, and participatory processes. Lessons learned from areas of historical coexistence between humans and large carnivores provide critical insights to design successful management strategies in areas of recent and future recolonisation by large carnivores.

**Abstract:**

Human-carnivore conflicts are a major conservation issue. As bears are expanding their range in Europe’s human-modified landscapes, it is increasingly important to understand, prevent, and address human-bear conflicts and evaluate mitigation strategies in areas of historical coexistence. Based on verified claims, we assessed costs, patterns, and drivers of bear damages in the relict Apennine brown bear population in the Abruzzo Lazio and Molise National Park (PNALM), central Italy. During 2005–2015, 203 ± 71 (SD) damage events were verified annually, equivalent to 75,987 ± 30,038 €/year paid for compensation. Most damages occurred in summer and fall, with livestock depredation, especially sheep and cattle calves, prevailing over other types of damages, with apiaries ranking second in costs of compensation. Transhumant livestock owners were less impacted than residential ones, and farms that adopted prevention measures loaned from the PNALM were less susceptible to bear damages. Livestock farms chronically damaged by bears represented 8 ± 3% of those annually impacted, corresponding to 24 ± 6% of compensation costs. Further improvements in the conflict mitigation policy adopted by the PNALM include integrated prevention, conditional compensation, and participatory processes. We discuss the implications of our study for Human-bear coexistence in broader contexts.

## 1. Introduction

Conservation conflicts are a major issue affecting wildlife populations and rural communities, globally [1]. In particular, conflicts between people and large carnivores are among the most challenging and complex to manage in conservation [2,3]. In the human-dominated landscapes of Europe, the development of rural economies and the conservation of large carnivore species are two aspects mandated by supranational legislation (EU Common Agricultural Policy; EU Habitats Directive; Bern Convention). Brown bears (Ursus arctos) are the most abundant large carnivore on the continent [4], permanently occupying 22 countries, stretching from western to south- and north-eastern Europe [4]. Bear populations in Europe have been increasing or stable in recent decades [4], also thanks to reintroductions [5]. With the ongoing bear expansion, Human-bear conflicts risk becoming increasingly exacerbated, especially in recently recolonised areas where local herding practices have changed and may no longer account for predator presence [4,6]. Such conflicts can also hinder the growth of endangered populations, due to cultural and social resistance to bear presence leading to high human-related mortality and counterbalancing conservation efforts [7].

Understanding, monitoring, and promptly intervening to address these conflicts is key for Human-bear coexistence. Although gaining insights into the mechanisms driving bear impacts on human activities is essential for developing effective mitigation strategies [6], there is still relatively little knowledge on these aspects (cf. [8]). Compensation schemes represent a common tool adopted to mitigate conflict and foster coexistence between humans and wildlife, both globally [9] and in Europe [8,10]. Such schemes entail reimbursing farmers for damages caused by predators, and are generally implemented ex-post (i.e., after the impacts occurred; [8]). Nonetheless, the effectiveness of these tools on their own to mitigate conflict remains subject of ongoing debate [9,11,12]. One way to improve the potential for conflict mitigation of compensation schemes has been through pairing them with the use of preventive measures (e.g., electric fences, livestock guarding dogs), thus aiming to reduce the actual impacts of the predators on farms [8,10,13]. Yet, albeit these efforts, the economic impacts of bear damage to human property continue to rise [8]. Recent estimates for the period 2005–2012 suggest that yearly compensation costs per individual bear, average at about € 1800 across Europe, varying largely between countries, independently of bear numbers [8]. In Italy, compensation costs paid for bear damages are particularly high, relative to other European bear populations, being lower only than Norway, Switzerland, and France [8]. Although wealthier countries tend to spend more in compensation, this does not necessarily translate in reduced bear impact or decreased compensation costs in the long term [8,14].

In Italy, the brown bear occurs in two separated populations, one on the Alps and the other on the central Apennines [15]. Both populations are strictly protected and target of extensive conservation efforts, especially in terms of damage compensation [16,17]. While the Alpine bear population has been reintroduced from Slovenian founders [18], Apennine bears (*U. a. marsicanus*) are the remnant of a relict, autochthonous, and isolated population of unique conservation value [19]. The remaining core of the Apennine bear population is found in the National Park of Abruzzo Lazio and Molise (PNALM) and contiguous areas [7] where, despite several decades of intense protection, the population has failed to recover or expand its range [7,20]. Although locally co-occurring with humans since historical times, Apennine bears remain critically endangered to date, with small population size, low genetic variability, inbreeding, and excessive human-related mortality representing the main threats [19,21,22,23]. Various social surveys have been conducted in the PNALM to explore local attitudes towards bears, documenting relatively positive views among the local people and their tolerance to coexist with bears [24,25,26]. The information currently available on the impact that Apennine bears have on the local economies, and their costs in terms of compensation, is limited to two preliminary studies conducted in the period 1988–2003 [27,28] and broad compilations at the European scale [8,16]. There is, therefore, a critical dearth of data on patterns of bear damages for a longer period, useful to assess trends and efficacy of compensation policies. This is problematic for two main reasons: first, this knowledge gap stymies innovative attempts addressed at mitigating local conflict, which to date continue to be a main hindrance to the expansion of the Apennine brown bear to suitable habitat beyond protected areas in the central Apennines [7,21]; second, given the historical coexistence between humans and bears within the PNALM, this lack of data prevents us from drawing valuable management insights to ameliorate Human-bear conflicts outside the park area and in human-dominated landscapes elsewhere [4,8].

This study aims to contribute towards knowledge of the impact of Apennine bears in their core distribution, through conducting a long-term (2005–2015) analysis of bear damages to various typologies of farms in the PNALM and its external buffer zone. In addition to evaluating the impact and financial dimension of the Human-bear conflict as indicated by compensation costs and their trend across time, we also investigated the potential drivers of brown bear damages at the local scale of analysis. In particular, we tested hypotheses concerning management, husbandry, and administrative factors likely affecting bear impact and its trend across time. Due to the long history of coexistence between humans and bears in the PNALM we believe our findings can be valuable to inform local and international conservation practice.

## 2. Materials and Methods

### 2.1. Study Area and Context

**Figure 1:**
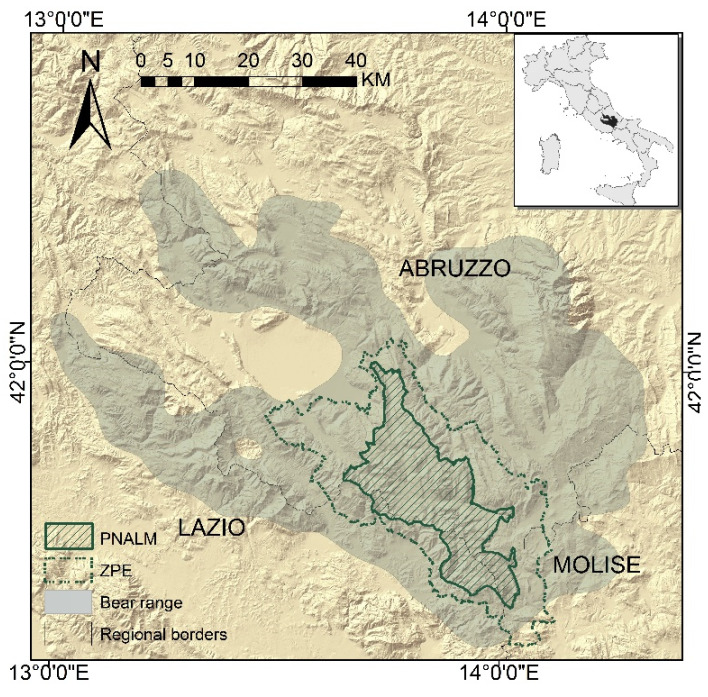
Location of the study area in central Italy (inset), comprising the National Park of Abruzzo Lazio and Molise (PNALM) and its external buffer area (ZPE), both within the core distribution of the relict Apennine brown bear population.

Our study area corresponds to the core range of the Apennine brown bear, including PNALM and its external buffer zone (ZPE), for a total of about 1294 km^2^ of the central Apennines (Figure 1). Elevation ranges from 400 to 2285 m and local temperature and rainfall patterns reflect the classic Mediterranean montane climate, with temperature varying widely between January and July (2–20 °C) and most of the rainfall occurring during spring and autumn. The winter months are generally characterised by snowfall and snow cover [29]. Beech (*Fagus sylvatica*) and, at lower altitudes, oak (*Quercus cerris* and *Q. pubescens*) forests cover about 60% of the study site, with subalpine meadows and grasslands covering about 22% [30]. Human population and road densities average 14.6 inhabitants/km^2^ and 1.1 km/km^2^, respectively [30]. Deforestation activities in the PNALM are strictly regulated by the Park Authority. Hunting is banned within the park but it is allowed in the ZPE [31]. Livestock husbandry, forestry, and tourism are key revenue sources for the local population, while agricultural activity is scarce and mostly confined to valley bottoms nearby human settlements. Traditional sheep herding practices [27] comprise relatively small flocks (only 13% with more than 300 heads) and close surveillance by a shepherd and several livestock guarding dogs (Abruzzo mastiff); the flocks are enclosed at nighttime within corrals, though these are not always predator-proof. The sheep grazing season at high elevation generally extends from June through October, but can be longer at lower altitudes. On the contrary, cattle and horses are left free ranging for most of the year and unprotected from predators including parturition. Although long-distance transhumance has almost disappeared from the area, several livestock owners seasonally move their flocks or herds at higher altitudes during spring and summer. To address conflict between humans and large carnivores, compensation to farmers in the PNALM was initiated by the Italian branch of the NGO World Wide Fund for Nature (WWF) in 1969. The Park Authority then took over in 1971, and has since been reimbursing claimed damages verified by the park personnel. In 1974, when the ZPE was established, compensation by the Park Authority was extended to this area. Claimed damages are verified by the park personnel within 24–48 h, and all compensation schemes adopted by the PNALM during the study period ensured 100% of the verified losses to farmers, plus an estimated amount corresponding to the lost productivity. Since 2000, the Park Authority has also been providing technical support and preventive tools (mostly wire and electric fences) on loan to local farmers, to promote the adoption of prevention measures.

The bear population has been estimated at 51 (95% CI = 47–66) bears in the core range [30] and was stable during our study period [32]. The bear active period extends from mid-March to the end of November/mid-December, though a few individuals may remain intermittently active throughout the winter period [33].

### 2.2. Data on Bear Impact

We compiled data on bear depredation events from claims verified by the Park Authority both in the park and its external buffer area from 2005–2015. Each claim generally corresponded to one bear attack. As an index of bear impact, we used the number of events per farm type per year and, for livestock farms, the number of depredated livestock heads by bear attack [34]. We recorded the recurrence of bear attacks per farm and year, and considered farms whose frequency of attack on an annual basis was equal to or greater than the 95th percentile as those suffering from chronic levels of bear impact (i.e., chronic farms [35]). For each bear depredation event, we also compiled the corresponding compensation costs paid by the Park Authority and tallied them by typology of farm and year. We did not account for yearly fluctuations in inflation or market price as these were considered negligible compared to the annual variation in compensation costs we reported.

We inventoried the number and type of farms registered in the study area in 2000 from the National Census of Agriculture [36], separately for the park and its external buffer area, assuming these did not change significantly during the study period. Livestock farms, however, were inventoried accessing the National Livestock database (BDN) relative to 2009 [37]. This included data on livestock number and composition, except for horses whose numbers were extracted from [36] as the BDN was only partly implemented at the time of the study. Information about apiaries and small animal farms (mostly poultry and rabbits) was obtained directly from PNALM’s 2010 databases, assuming their numbers did not change significantly during our study. To gather information on transhumant livestock owners (i.e., those that seasonally move flocks or herds across different townships), we compiled at the township level all official permits released by the National Health System. Currently, only a minority of livestock farms move long distances during transhumance, and most of them comprise farmers permanently residing within the study area who move their herds to higher altitudes during summer.

With the exclusion of livestock guarding dogs, traditionally adopted by most local farmers, we then finally compiled information on farms that received preventive tools on loan from the Park Authority. While some farms were self-sufficient in equipping themselves with prevention measures, no complete inventory of such farms is currently available. Therefore, in our analysis we contrast farms that received vs. those that did not receive prevention incentives from the Park Authority, where the latter may include an unknown proportion of farms already equipped with preventive measures.

### 2.3. Data Analysis

We developed two classes of models: (a) those investigating patterns of bear attacks on all typologies of farms, including livestock, small animals (mostly poultry and rabbits), beehives, and cultivations (crops, orchards, and fruit trees); and (b) those specifically investigating bear attacks on livestock farms. In particular, we expected that bear damages would be distributed across the various types of farms depending on the profitability and seasonal availability of their production (H1). Management-wise, we also expected that farms within the park area, compared to farms in the ZPE, would be on average better equipped to prevent bear attacks as a result of higher conservation attention by the park administration (H2) and, specifically, that farms who benefitted from prevention incentives from the Park Authority would also be less susceptible to bear damages (H3). We finally expected that, on a seasonal basis, bear damages would increase during the hyperphagic season (H4), and that bear damages would tend to decrease throughout the study period on an annual basis (H5) due to a combination of: increasing number of farms who benefitted from prevention measures, mitigation measures adaptively improved by the side of the Park Authority, and an apparently stable bear population [33]. Therefore, predictors included in this class of models comprised: Type of production (cultivation, beehives, livestock, other farm animals), Management zone (park vs. external buffer zone), Prevention (i.e., if a given farm received preventive tools on loan from the Park Authority in previous years), Season (Spring, Early summer, Late summer, Autumn, Winter [38]), and Year. In the second class of models, related to pattens of bear damages to livestock farms, in addition to the hypotheses H2, H3, and H5 above, we also expected that sheep and goats, being more abundant and accessible than other livestock species, would be more frequently depredated by bears (H6). We also expected that livestock depredations would be more frequent during summer months when livestock grazes on pastures, but also that an interaction between season and livestock species would account for temporal differences in the accessibility of some species (e.g., calves and foals; H7). Finally, we expected that compared to resident livestock farmers, transhumant ones would be more exposed to bear attacks (H8) due to their less effective prevention in temporary summer grazing allotments. On this note, predictors of this class of models also comprised Livestock species (cattle, horses, sheep, and goats pooled) and Farm residency (resident vs. transhumant). We did not include in the model the density of the different livestock species because marked differences in their husbandry and surveillance methods (see Study area) would make it a poor indicator of their true accessibility to bears.

To test the above hypotheses, we developed generalised linear models (GLM) in R (version 4.0.3; [39]) using the number of bear attacks as a response variable and the above-mentioned factors as predictors. Starting from a saturated model, we then used the R package MuMIn [40] to compare all possible submodels through dredging and finally performed model selection using the sample-size corrected Akaike’s Information Criterion (AICc) [41]. We then adopted multi-model inference averaging estimates based on model weights retaining models within AIC ≤ 2 from the best supported model [41]. Averaged coefficients in the final models were deemed significant if their 95% confidence interval (CI) did not include zero. We finally used the Nagelkerke Pseudo-R^2^ [42] to assess how each of the averaged models fit the data. Using R [39], we also conducted ad hoc tests (i.e., G-test, Kolmogorov-Smirnov test, Mann-Whitney U-test, Spearman’s correlation) to test specific hypotheses about distribution and trends of subsets of data. Average values were reported as mean values ± standard deviation (SD).

## 3. Results

### 3.1. Overall Bear Impact and Compensation Costs

From 2005 to 2015, an average of 203 ± 71 attacks by bears were verified by the park personnel each year, corresponding to 75,987 ± 30,038 €/year paid for compensation (Table 1). Excluding structures, 268 cultivated crops, 240 livestock, 184 small animal, and 56 honey farms were cumulatively damaged by bears in the PNALM during the study period.

On average, 6 ± 2% of livestock farms, and 20 ± 10% of honey farms active in the study area suffered from verified bear damages on an annual basis.

According to our final averaged model, the number of bear depredations was primarily affected by Type of production, Management zone, Prevention, Season, and Year, even though the latter did not yield a significant coefficient (Table A1 in Appendix A). Specifically, compared to beehives, bear depredations were higher in livestock farms (β = 0.503; 95%CI: 0.151–0.852) and, accounting for an interaction between Type of production and Season, also for cultivations in early and late summer (1.247 ≤ β ≤ 1.406; all significant) and small animal farms from spring to late summer (1.032 ≤ β ≤ 2.001; all significant) (Table A1), thereby supporting our H1. Accordingly, livestock depredations ranked first in terms of compensation (59.5%) and, even though cultivations and small animal farms were more frequently affected by bears compared to beehives, the latter ranked second in terms of compensation costs (21.8%) (Table 1).

**Figure 2:**
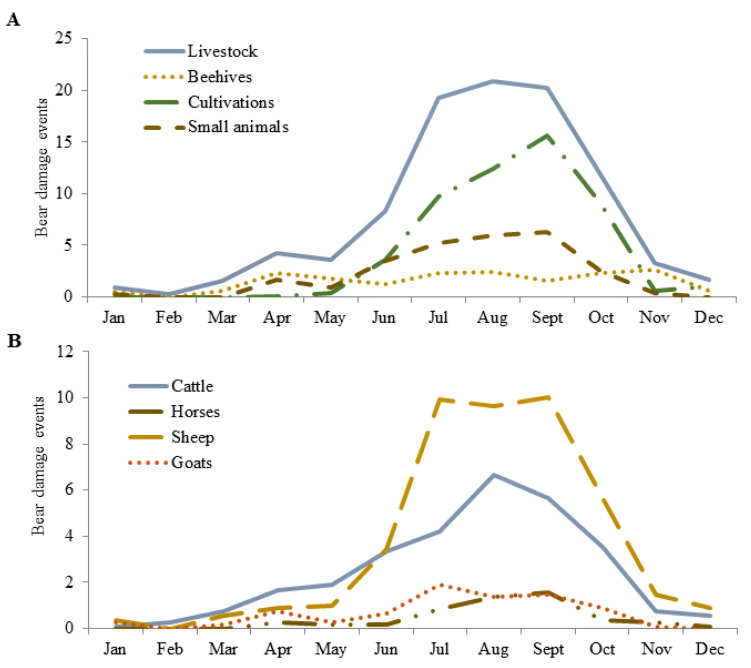
Distribution of bear damages by month, including: (**A**) all types of damage except Structures (*n* = 2232 damage verification records); (**B**) damages to livestock only (*n* = 983, excluding small animals) in the Abruzzo Lazio and Molise National Park (central Italy, 2005–2015).

**Figure 3:**
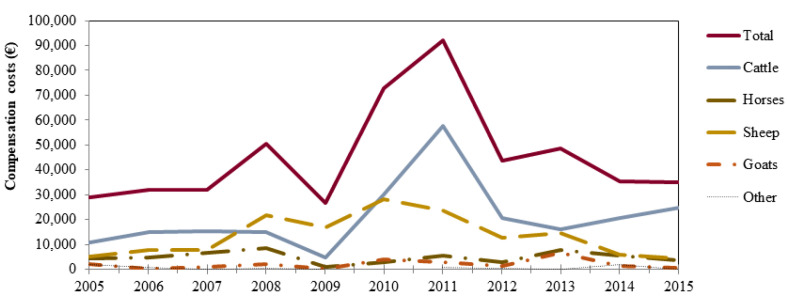
Distribution of compensation costs on an annual basis concerning all types of damages (*n* = 2232 damage verification records) caused by bears in the Abruzzo Lazio and Molise National Park, Central Italy (2005–2015).

Compared to the park area, farms active in the ZPE suffered a higher occurrence of bear damages (β = 0.560; 95% CI: 0.460–0.660), corresponding to 65 ± 9% of the damages (65 ± 11% of compensation costs). This is in line with our H2, even though bear damages in the two management units were distributed in proportion to their relative extension (G_adj_ = 63.14, df = 9, *p* < 0.01). Farms that adopted prevention measures incentivised by the Park Authority appeared less susceptible to bear attacks compared to the other impacted farms (β = −1.786; 95%CI: −1.970–−1.604), thereby confirming H3. A seasonal effect also confirmed H4, with bear damages being highest during late summer and autumn, and lower in the other seasons (−1.372 ≤ β ≤ −0.887; all significant). Accordingly, bear damages were not uniformly distributed on a monthly basis (K-S 1-sample: 0.18 ≤ D ≤ 0.39, *p* < 0.01; Figure 2A). Although a Year term was included in the second ranking model, the overall fit of the model did not improve and its coefficient was not significant, thus no annual trend in bear damages was apparent throughout the study period, refuting our H5. Both the number of depredations and the corresponding compensation costs, however, varied markedly from year to year (Table 1), and compensation costs peaked in 2011 due to concomitant increase in bear damages to both livestock and beehives (Figure 3).

### 3.2. Impact on Livestock

From 2005 to 2015, an average of 96 ± 34 livestock depredations by bears were verified by the park personnel each year, corresponding to 45,221 ± 19,383 €/year paid for compensation (Table 1). Bear depredations afflicted on average 1.1% (horses)–7.3% (sheep) of the registered livestock farms in the study area, and losses compounded to 0.3% (goats)–0.7% (cattle) of livestock heads per year (Table 2). According to our final, averaged model, the number of bear attacks on livestock was affected by Livestock species, Management zone, Prevention, Farm residency, Season, and Year, even though the latter did not improve the fit of the model nor yielded a significant coefficient (Table A2). Specifically, compared to cattle, bear depredations were more frequent on sheep and goats (β = 0.551; 95%CI: 0.181–0.911), and less frequent on horses (β = −1.253; 95%CI: −2.205–−0.391), thereby supporting our H6. Accordingly, bear attacks on sheep comprised 45.8% of verified bear attacks on livestock (30 ± 14% of compensation costs), with an average of 44 ± 22 depredation events per year ($\bar{x}\;$= 91 ± 48 sheep heads/year) (Table A3). Verified attacks on cattle, however, ranked first in terms of compensation costs (45 ± 15%), comprising an average of 29 ± 12 depredation events per year ($\bar{x}\;$= 32 ± 14 cattle heads/year; Table A3). Based on the proportions of livestock species available in the PNALM, bear attacks appeared to be preferentially targeted at cattle and occurred less than expected on sheep (G_adj_ = 102.64, df = 3, *p* < 0.01).

Compared to livestock farms residing within the park area, livestock owners grazing in the ZPE suffered higher bear depredations (β = 0.379; 95%CI: 0.223–0.535), confirming H2. In addition, livestock owners who loaned prevention measures from the Park Authority were less susceptible to bear attacks than the other livestock owners impacted by bears (β = −1.263; 95%CI: −1.577–−0.948), in line with our H3. Contrary to our hypothesis (H8), however, bear depredations were lower for transhumant (β = −0.883; 95%CI: −1.125–−0.638) than resident livestock farms. On a seasonal basis, compared to autumn, bear depredations on livestock were expectedly lower in winter (β = −1.782; 95%CI: −3.151–−0.297) and highest in late summer (β = 0.703; 95%CI: 0.321–1.062), supporting H7; in addition, an interaction between Season and Livestock species revealed an even lower chance of sheep and goats to be predated by bears in spring (β = −0.516; 95%CI: −1.596–−0.201) compared to cattle. Accordingly, bear depredations on livestock were not uniformly distributed on a monthly basis (K-S 1-sample, 0.16 ≤ D ≤ 0.32, 0.01 ≤ *p* ≤ 0.05), with depredations on cattle increasing steadily during spring months (Figure 2B). Upon attack on livestock, bears usually depredated a single head and, whereas adult sheep and goats were the most frequent target, calves and juvenile horses appeared to be the age classes more frequently attacked (Table 3). Although no annual trend in bear damages to livestock was apparent throughout the study period (Table A2 in Appendix A), bear damages to cattle showed a peak in 2011, thereby contributing to a marked increase in the overall compensation costs in that year (Figure 3).

### 3.3. Recurrence of Attack and Chronic Farms

On a yearly basis, the number of farms affected by bears averaged from 6 (±4) to 49 (±14), representing annually 1–7% of all active farms (Table 4). We did not reveal annual trends in the number of farms damaged by bears for any typology (K-S 1-sample: 0.13 ≤ D ≤ 0.33, *p* > 0.05). Replicates of bear attacks by farm ranged 1–21 per year, with highest recurrence reported in livestock and honey farms (Table 4). Livestock farms chronically damaged by bears represented 8 ± 3% of the total affected on a yearly basis, averaging 3.7 ±1.3 farms/year and corresponding to 29 ± 7% and 24 ± 6% of depredation events and annual compensation costs, respectively. Livestock farms were the only ones suffering chronic levels of bear damages consistently each year, though no trends were revealed in their numbers throughout the study period (K-S 1-sample: D = 1.18, *p* > 0.05). However, only sheep farms suffered chronic depredation levels consistently each year, with an average of 1.9 ± 0.7 chronic farms per year suffering from 4 to 16 bear attacks per year; these sheep farms alone, represent 10 ± 3% of all bear-impacted sheep farms, and respond to 30 ± 14% of both overall sheep depredations and compensation costs.

### 3.4. Incentivised Prevention

In total, from 2005 to 2015, the PNALM authority loaned preventive tools for use to 299 farms ($\bar{x}\;$= 21 ± 17 farms/year). These mostly involved electric fences (86% *n* = 240) and related accessories (3%, *n* = 7), or wire fences and gates (11%, *n* = 31). The majority of incentives (69 ± 15% per year) benefitted farms with multiple production, whereas 19 ± 16% involved livestock, 10 ± 8% beehives, and 2 ± 3% agricultural farms. Loans by the Park Authority increased at a rate of 25.9 new interventions per year (R^2^ = 0.95, *p* = 0.03) throughout the study period. Nevertheless, a rather small proportion of farms that suffered bear damages in the previous years benefitted from such loans (livestock: 5 ± 5%; beehives: 23 ± 26%), and the number of farms incentivised in a given year was not correlated with the number of verified bear damages neither in the same (r_s_ = −0.37, *n* = 11, *p* > 0.05) nor in any of the previous two years (0.14 ≤ r_s_ ≤ 0.46, 9 ≤ *n* ≤ 10, *p* > 0.05). Similarly, farms that suffered chronic levels of bear damage in the previous years were not more likely to receive prevention measures from the Park Authority compared to those claiming low to moderate levels (0.32 ≤ G_adj_ ≤ 3.07, df = 1, *p* > 0.05); cumulatively, the latter represented 88.2 ± 13% of the farms that loaned prevention measures, a figure that raises to 95 ± 6% including farms that did not suffer from any bear damages in the previous years.

## 4. Discussion

In this study, we aimed to describe the extent of impact on human activities by a relict, small and imperiled bear population living at relatively high density (i.e., 39.7 bears/1000 km^2^ [30]) in a human-modified landscape. This knowledge is increasingly relevant to support current and future conservation programs in the prospects of bear population growth and expansion [13,19,43]. Moreover, understanding the underlying mechanisms of the management interface between humans and bears in this historical stronghold would benefit not only the sustained conservation of the Apennine brown bear, but also Human-bear coexistence in other similar contexts [4,8].

Overall, we found that in the PNALM, compensation costed an average of 75,987 € per year, or 1490 ± 589 €/bear/year based on the 2011 population estimate [30]. In comparison, this is about 55% lower than the compensation costs sustained by the same park for wolf depredations [35], and corresponds to a significant share of the annual budget available to the PNALM for conservation actions. Moreover, the costs we computed should be interpreted as minimum estimates, as they do not include the extra costs of prevention measures; although these costs could not be compiled for this study, the raw material to build electric fences provided by the Park Authority to farmers from 2010 to 2014 during the LIFE NATURE project Arctos (http://www.life-arctos.it/, accessed on 12 April 2021) costed 68,129 € (13,626 €/year). These findings confirm that bear damages in the PNALM rank relatively high compared to other European countries [8,17]. Although this indeed reflects the high density of farms and bears within the PNALM, it confirms the global pattern according to which wealthier countries place more institutional attention to compensation [17]. These costly compensation programs, however, do not necessarily correspond to enhanced prevention of bear damages, may jeopardize their own economic sustainability in the long term, and may also deduct from limited funds that could be used for other conservation initiatives. On this note, the average annual compensation costs we estimated in the PNALM are higher than those reported in the period 1998–2003 (59,422 €/year; [27]). In addition, during our study, compensation costs varied markedly from year to year, peaking in 2011 as high as 151,154 € (2964 €/bear/year) due to a synergistic increase in both cattle and beehives bear damages. This should not be regarded as a rare or occasional event, because following mast years, or when climatic conditions may significantly reduce the availability of natural foods, bears may be forced to make increased use of anthropogenic foods [44,45,46,47]. Therefore, any action addressed at reducing the occurrence of bear damages, and at making more efficient use of limited conservation funds, would further enhance the effectiveness and economic sustainability of the current conflict mitigation policy.

With the exception of some year-to-year variability, we did not detect a decreasing trend in bear damages across the study period, which is contrary to our prediction (H5) and to what would be expected under forms of adaptive management of compensation schemes [6,48,49]. Nonetheless, the lack of increasing trends of bear impact on local farms could still be viewed as a measure of success, though costly, of the local conflict mitigation management. Moreover, these findings may also reflect the park’s management approach, which is less focused on reducing compensation costs and more geared towards making farmers comfortable with bear presence and Park Authorities, as to improve tolerance and erode motivation for retaliatory killing of bears and other wildlife. It should be noted, however, that programs that aim to improve tolerance by only paying compensation can perpetuate negative attitudes and escalate conflicts over large carnivore conservation [10,50]; instead, by making effective use of prevention funds, lower compensation costs may actually correspond to positive attitudes and functional management of Human-bear conflicts [8]. In this perspective, even though we could not confirm our adaptive management hypothesis (H5), the Park Authority attempted adaptive management in compensation through the years by refining compensation schemes and increasing incentives for the adoption of prevention measures. Compared to the period 1998–2003 [27], in addition to the ever increasing number of farms that received prevention incentives from the park, our findings also indicate a decrease in compensation for bear damages to beehives (16,572 €/year in our study vs. 23,452 €/year in 1998–2003) and a reduced time farmers had to wait from claim to compensation (an average of 5 months in our study vs. 3 months—3 years in 1998–2003); the latter, in particular, originates from a simplification of the bureaucratic procedure to activate compensation. While these management aspects are central to coexistence, they remain poorly implemented and administered globally [9]. The PNALM’s approach can therefore be viewed as having been rather successful at its goal, considering the positive attitudes towards bears documented locally [24,25] and a 53.2% decrease in the number of bears illegally killed in the past decade compared to previous years [51]. This is notable especially considering that the park area features as many as 93 livestock farms/100 km^2^, a minimum of 4 honey farms/100 km^2^, and numerous small, agricultural farms and cultivations scattered across valley bottoms and in the periphery of small mountain villages. Yet, we maintain that additional efforts and further improvements can be made to render compensation programs less costly, more efficient, and more integrated into a broader conflict mitigation strategy (see below).

The patterns of bear damages and their possible drivers disclosed in this study conveys valuable information for further refining and correcting the compensation programs in place during the study period. In particular, our findings show that bear damages occurred throughout the bear-active period and increased from spring to late summer and autumn for all types of farms, but especially for livestock farms. This confirms trends documented by studies in other regions at similar latitudes and in similar ecological settings (e.g., Greece: [52]; Spain: [53,54]). Specifically, similar to many other European countries [8], among all types of damages made by bear in the PNALM, livestock depredations were the most frequent and economically impacting (60% of annual compensation costs paid for bear damages). Damages to cultivations and small farm animals followed in terms of occurrence, but those on beehives ranked second in terms of compensation costs and were more evenly distributed throughout the bear-active period. This is also in line with damage patterns observed in broader European contexts [17], thus underscoring these types of impacts as main sources of Human-bear conflicts requiring particular management attention [4,8]. Although the damages to cultivations and small farm animals were, overall, less expensive, their importance towards fostering coexistence should nevertheless not be overlooked, as these damages can still be impactful on local economies and are also more difficult to prevent using traditional methods. Crucially, we also found that the incentives loaned by the Park Authority for prevention corresponded to a marked decrease of susceptibility to bear damages of the various type of farms, including livestock farms, emphasising the importance of prevention in mitigating bear impact and reducing compensation costs in the long term. Therefore, while the objective of our analyses was not to assess the individual performance of the different kinds of preventive measures, our overall results also positively feed into ongoing debates on the role of prevention for coexistence [55,56,57].

Our study has also shed light on the relations between bears and transhumant pastoralists, for which there is a dearth of data (cf. [56]). Contrary to our initial hypothesis, we found that bear attacks on livestock occurred less frequently in transhumant farms compared to residential ones. Possible non-mutually exclusive explanations for this include that: (i) bears, by virtue of learning, visit residential farms more often within their home ranges, whereas the discontinuity and an overall shorter presence of the transhumant ones on pastures offer limited learning opportunities; (ii) transhumant livestock owners generally graze their flocks at higher altitudes during summer, far from forested areas where core areas of bear home ranges are located, thereby reducing encounter rates with bears; or (iii) transhumant shepherds are better equipped to confront large carnivores and adopt husbandry and surveillance techniques particularly apt to reduce the losses during the relatively short summer grazing season. More in-depth field investigations at the scale of the single farm would be necessary to unveil these details; nevertheless, this suggests that locally transhumant livestock owners, possibly due to their long experience with bears and other large predators, are also in a position to effectively deal with bears and minimise their impact in the area.

Although we found that the largest share of depredated livestock by bears were sheep, this is explained by their higher occurrence throughout the PNALM [27]. In fact, compared to their relative availability, sheep were depredated by bears in lower proportions, whereas cattle were positively selected. This is likely a consequence of cattle (and horses) left free ranging on pastures and with little to no surveillance throughout the grazing months, including birthing, when unprotected calves and foals are particularly vulnerable to bear and wolf predation [27,37]. Traditionally, livestock guarding dogs have always been used in the area, though mostly with sheep and not with cattle or horses. On this note, providing cattle and horse farmers with prevention tools adequate to protect calves and foals from large predators [58,59,60] could go a long way towards reducing bear impact on these categories, and hence in reducing compensation costs, as cattle ranked first with a share of 20,941 ± 13,309 € per year. In contrast, preventing damages to cultivations is more difficult as these are widespread and often comprise crops and backyard fruit trees or domestic orchards. In these cases, a relevant deterrent would be to remove potential anthropogenic foods that attract bears to villages where they could then have easy access to cultivations, such as orchards, fruit trees, and small animals such as poultry. Similar to other forms of supplemental feeding, accessibility of anthropogenic foods to bears is a poor practice in an area hosting a relatively high density of bears and a diffuse presence of human activities, such as in the PNALM. In fact, under these conditions, a few habituated and food-conditioned bears have learnt to frequent small mountain villages in the PNALM, and although attempts have been made to proactively discourage them through negative conditioning, results have been inconclusive so far [61]. In the period 1998–2003, substantial bear damages to beehives and poultry were actually perpetuated by only two nuisance bears, cumulatively accounting for about 35% of yearly compensation costs in the PNALM [27]. Encouraging feeding by bears on non-natural foods by their recurrent availability may alter their foraging behaviour and reinforce their dependency on convenient anthropogenic foods, bearing negative implications for the further development of Human-bear conflicts [62,63]. Whereas proper habitat management is key to ensure the quality and long-term productivity of natural foods in the PNALM ecosystem [37], effective information, door-to-door education campaigns, and technical support need to be provided to local residents in order to encourage reducing the accessibility of anthropogenic foods to bears [64,65,66] (but see [67]).

## 5. Conclusions

This study has contributed an original glimpse into the current costs and management dynamics of the historical Human-bear coexistence in the PNALM. There are several implications that follow from this investigation, concerning both the local and more global contexts of Human-bear coexistence. First, compared to the large number of farms active in the PNALM, the rather small proportion of farms that suffer from bear attacks is indicative that the cultural and technical understanding of how to coexist with bears is widespread among local farmers, including transhumant ones. It also indicates that the added labour required to effectively implement prevention measures is functionally integrated into their management and husbandry practices. This is likely due to a long time of coexistence with bears and to the widespread perception that bears benefit farmers’ business by fostering locally a tourism-based economy (Glickman et al., unpublished data). Second, we find there is ample room for improvement in terms of prevention and compensation policy. In particular, livestock farms that suffer from chronic levels of damage should become the priority of any further prevention campaign. We revealed that the annual mean of recurrence of bear attacks on the same livestock farm exceeded 1 for all livestock species, and was as high as 16 per year in sheep farms. Each year, from 2 to 6 livestock farms suffered chronic levels of bear attacks, averaging about 8 attacks per year and corresponding to 24% of the total compensation costs. The proportion of such farms did not decrease during the study period, nor were these farms more likely to receive prevention incentives compared to those that claimed lower or no levels of bear impact. Therefore, a more targeted selection of the farms eligible for incentivised prevention by the Park Authority could go a long way towards decreasing chronic levels of bear impact and hence reducing compensation costs in the long term [68]. Monitoring and analysing distribution and recurrence of bear attacks at the farm level on a yearly basis, as we did in this study, should become a relevant and permanent component of any adaptive strategy meant to mitigate Human-bear conflict in the park. In addition to this, completing an inventory of all active farms in the PNALM, including a functional description of the adopted prevention tools and methods, would be essential to more effectively target prevention incentives by the Park Authority. Monitoring the effectiveness of prevention measures at the level of single farms, as well as the distribution and recurrence of bear attacks across the years, would allow to adaptively improve the overall conflict mitigation policy adopted by the park [6,69,70]. In this perspective, given the elevated number of farms that already loaned prevention measures from the park and those who autonomously acquired them, it would be possible for the Park Authority to swiftly shift to a compensation program that is conditional on prevention [8]. The PNALM compensation policy would also benefit from becoming fully integrated into a participatory process (e.g., [71,72]), sharing with local stakeholders management goals and actions, and co-developing solutions to benefit from the presence of bears and other wildlife [65].

Implications of our work extend beyond the boundaries of the PNALM. The policies and approaches implemented by the Park Authority enlighten possible ways to address Human-bear coexistence in human-dominated landscapes more in general. Concurrently, however, our findings also reinforce the notion that mitigation of Human-bear conflicts is costly and hardly affordable in less affluent countries where many small and endangered Ursid populations reside [73]. It is therefore necessary to develop and assess less costly and more efficient strategies, increasingly integrated with preventive measures, adequate livestock husbandry techniques, participatory processes, monitoring, and adaptive management. In addition, specifically concerning protected areas, based on our experience we believe a relevant issue revolves around what density of livestock farms should be allowed, where, and under what conditions if we aim to ensure a functional coexistence between humans and bears. In recent decades, the livestock sector in the PNALM witnessed a marked increase in the number of cattle and horse farms and a concomitant reduction in the sheep-oriented livestock production [27,35]. While traditional sheep husbandry techniques are being replaced by more profitable and less predator-compatible forms of cattle and horses raising [35], the latter contribute to proportionally higher levels of bear damages and compensation costs. With this in mind, it is perhaps unsurprising that bear damage compensation is expensive in the PNALM, which hosts an average of 25 cattle farms/100 km^2^ and 344 cattle heads/100 km^2^. At least within protected areas, bear conservation planning should not only consider prevention and compensation tools, but also discourage an excessive number of livestock farms, as well as livestock species and husbandry techniques, which are incompatible with bear populations thriving at an ecological equilibrium density. Such understandings are particularly relevant in view of the ongoing bear expansion across Europe’s densely populations regions, which heralds increased Human-bear interactions in the forthcoming decades in contexts similar to the one investigated here [4,5].

## Figures and Tables

**Table 1:** Verified damages caused by bears, and corresponding compensation costs, in the National Park of Abruzzo Lazio and Molise, central Italy (2005–2015). Data compiled from 2232 verified claims.

Year	Damage Events						Compensation Costs					
	Total	Livestock	Beehives	Cultivations	Small Animals	Structures	Total	Livestock	Beehives	Cultivations	Small Animals	Structures
2005	83	69	1	9	4	–	37,075	29,060	5460	1724	831	–
2006	205	88	26	41	41	9	61,101	32,087	19,497	4481	4546	490
2007	147	71	5	27	37	7	50,949	32,005	4297	10,898	3437	312
2008	272	144	34	69	22	3	92,666	50,459	26,319	9092	3198	3597
2009	152	75	13	39	25	–	56,566	26,565	19,893	5990	4118	–
2010	274	139	16	66	39	14	103,555	72,678	12,041	8565	8341	1930
2011	317	155	37	81	26	18	151,154	91,891	37,877	10,788	4498	6101
2012	279	97	26	102	37	17	85,931	43,570	21,201	12,702	6628	1830
2013	198	105	9	34	30	20	66,357	48,741	4391	5810	4715	2700
2014	183	66	24	65	22	6	71,394	35,508	19,301	10,430	5195	960
2015	122	48	9	44	10	11	59,111	34,865	12,012	7190	3044	2000
*mean*	*203*	*96*	*18*	*52*	*27*	*10*	*75,987*	*45,221*	*16,572*	*7970*	*4414*	*1811*
*(±SD)*	*71*	*34*	*11*	*25*	*11*	*7*	*30,039*	*19,383*	*9822*	*3118*	*1866*	*1751*

**Table 2:** Impact of bear depredations on livestock in terms of affected farms and depredated livestock heads per year, averaged across years. Data compiled from 983 verified livestock damage claims in the Abruzzo Lazio and Molise National Park, central Italy (2005–2015).

Species	Livestock Farms Active in the PNALM ^1^		Livestock Affected by Bear Depredations			
	Total No. Farms	Total No. Livestock Heads	Farms		Livestock Heads	
			$\bar{\chi }\pm SD$	% ^2^	$\bar{\chi }\pm SD$	% ^2^
Sheep	282	17,583	21 ± 9	7.3 ± 3.0	80 ± 46	0.5 ± 0.3
Cattle	328	4445	20 ± 7	6.2 ± 2.1	32 ± 14	0.7 ± 0.3
Goats	116	4546	6 ± 5	5.1 ± 4.1	12 ± 11	0.3 ± 0.2
Horses	473	2090	5 ± 2	1.1 ± 0.5	9 ± 5	0.4 ± 0.2

^1^ Data obtained from [36,37] and grazing leasing forms for 2010 compiled at the township level. ^2^ Proportion relative to farms (number of livestock heads) in the study area.

**Table 3:** Outcomes of bear depredations on livestock and their age distribution (months ± SD) as assessed by park veterinarians upon verification of claimed attacks. Data compiled from 983 verified livestock damage claims in the Abruzzo Lazio and Molise National Park (central Italy, 2005–2015).

	No Depredated Heads/Bear Attack		Age-Class (Months)		
	Min–Max	Median	<2	2–24	>24
Cattle (*n* = 322)	1–3	1	51 ± 27%	34 ± 22%	16 ± 7%
Horses (*n* = 94)	1–2	1	23 ± 20%	62 ± 25%	16 ± 18%
Sheep (*n* = 481)	1–16	1	1 ± 1%	4 ± 5%	95 ± 5%
Goats (*n* = 86)	1–7	1	4 ± 10%	4 ± 7%	92 ± 12%

**Table 4:** Number and type of farms affected by bear damages, replicates of bear attacks, and characteristics of farms that suffered chronic levels of bear attacks. Data from 2232 verified claims in the Abruzzo Lazio and Molise National Park, central Italy (2005–2015).

Type of Damage	No. of Damaged Farms by Bears per Year		Recurrence of Attack per Farm per Year ^1^		Farms with Chronic Levels of Damage per Year				
	Mean (±SD)	% ^2^	Min–Max	Mean (±SD)	Min–Max	Mean (±SD)	% ^3^	Depredation Events	
								Min–Max	Mean (±SD)
Livestock ^4^	49 ± 14	5 ± 2%	1–21	1.9 ± 0.3	2–6	3.7 ± 1.3	8 ± 3	3–21	7.7 ± 2.9
Sheep	21 ± 8	7 ± 4%	1–16	2.0 ± 0.4	1–3	1.9 ± 0.7	10 ± 3	4–16	6.6 ± 2.2
Cattle	20 ± 7	6 ± 2%	1–11	1.4 ± 0.1	0–5	1.7 ± 1.3	8 ± 5	3–11	3.7 ± 0.6
Goats	6 ± 5	3 ± 4%	1–4	1.4 ± 0.4	0–1	0.1 ± 0.3	1 ± 3	4	4.0
Horses	5 ± 2	1 ± 1%	1–8	1.7 ± 0.8	0–1	0.4 ± 0.5	8 ±12	4–8	6.3 ± 1.5
Cultivations	43 ± 18	-	1–6	1.1 ± 0.1	0–4	1.2 ± 1.5	2 ±3	4–6	4.3 ± 0.4
Small animals	24 ± 10	-	1–4	1.1 ± 0.1	0–1	0.2 ± 0.4	1 ±2	4	4.0
Beehives	10 ± 5	4 ± 3%	1–10	1.7 ± 0.4	0–3	0.8 ± 1.0	7 ±9	4–10	6.0 ± 2.2
Structures	6 ± 4	-	1–2	1.0 ± 0.4	-	-	-	-	-

^1^ No. of bear damage events per farm per year. ^2^ Proportion relative to all farms active in the PNLAM (including the external buffer area); ^3^ Proportion relative to all farms of the same type affected by bear attacks; ^4^ Including damages to unidentified livestock.

## Data Availability

The data presented in this study are available on request from the corresponding author. The data are not publicly available due to privacy restrictions.

## Appendix A

**Table A1:** Generalized linear model selection, and corresponding model coefficients, to investigate patterns of damages caused by bears to farms in the National Park of Abruzzo Lazio and Molise, central Italy, 2005–2015. Only candidate models with ∆AICc ≤ 2 are shown. Model coefficients were estimated through full averaging limited to models with ∆AICc ≤ 2. R^2^: Nagelkerke Pseudo-R^2^ (averaged models only); K: number of estimable parameters; LL: log-likelihood; AICc: Akaike information criterion adjusted for small sample sizes; ∆AICc = (AICc) − (AICc)min; w: Akaike weight.

Model Structure	R^2^	K	LL	AICc	∆AICc	w
Type of production + Mgt zone + Prevention + Season + Season*Type of production	0.958	6	−1061.73	2170.3	0.00	0.734
Type of production + Mgt zone + Prevention + Season + Year + Season*Type of production	0.958	7	−1061.61	2172.3	2.03	0.266
**Model Coefficients**	**β**	**SE**	**95% Confidence Interval**			
			**Lower**		**Upper**	
(intercept)	3.160	8.925	0.773		1.416	
Type of production ^1^ (Small animals)	−1.090	0.280	−1.638		−0.543	
Type of production (Cultivations)	0.006	0.191	−0.368		0.378	
Type of production (Livestock)	0.503	0.179	0.151		0.852	
Mgt zone (ZPE) ^2^	0.560	0.051	0.460		0.660	
Prevention (Yes) ^3^	−1.786	0.094	−1.970		−1.604	
Season (Spring) ^4^	−0.887	0.252	−1.383		−0.395	
Season (Early summer)	−1.020	0.266	−1.541		−0.500	
Season (Late summer)	−0.371	0.244	−0.852		0.106	
Season (Winter)	−1.372	0.599	−2.546		−0.199	
Year	−0.001	0.004	−0.008		0.075	
Season (Spring)*Type of production (Small animals)	1.032	0.418	0.213		1.853	
Season (Early summer)*Type of production (Small animals)	2.001	0.371	1.275		2.728	
Season (Late summer)*Type of production (Small animals)	1.700	0.349	1.018		2.385	
Season (Winter)*Type of production (Small animals)	2.147	1.192	−0.193		4.480	
Season (Spring)*Type of production (Cultivations)	−15.19	491.94	−980.93		950.56	
Season (Early summer)*Type of production (Cultivations)	1.247	0.301	0.657		1.838	
Season (Late summer)*Type of production (Cultivations)	1.406	0.273	0.873		1.941	
Season (Winter)*Type of production (Cultivations)	1.320	0.755	−0.157		2.802	
Season (Spring)*Type of production (Livestock)	0.437	0.288	−0.124		1.003	
Season (Early summer)*Type of production (Livestock)	1.551	0.285	0.994		2.109	
Season (Late summer)*Type of production (Livestock)	1.325	0.262	0.813		1.841	
Season (Winter)*Type of production (Livestock)	−0.776	0.690	−2.128		0.577	
Season (Spring)*Type of production (Livestock)	0.437	0.288	−0.124		1.003	

^1^ Reference: Beehives; ^2^ Reference: Park; ^3^ Reference: No prevention; ^4^ Reference: Autumn. *: interaction (multiplicative effect).

**Table A2:** Generalized linear model selection, and corresponding model coefficients, to investigate patterns of damages caused by bears to livestock farms in the National Park of Abruzzo Lazio and Molise, central Italy, 2005–2015. Only candidate models with ∆AICc ≤ 2 are shown. Model coefficients were estimated through full averaging limited to models with ∆AICc ≤ 2. R^2^: Nagelkerke Pseudo-R^2^ (averaged models only); K: number of estimable parameters; LL: log-likelihood; AICc: Akaike information criterion adjusted for small sample sizes; ∆AICc = (AICc) − (AICc)min; w: Akaike weight.

Model Structure	R^2^	K	LL	AICc	∆AICc	w
Livestock species + Mgt zone + Prevention + Farm residency + Season + Season*Livestock species	0.779	7	−628.604	1295.8	0.00	0.394
Livestock species + Mgt zone + Prevention + Farm residency + Season	0.638	6	−637.822	1296.4	0.67	0.282
Livestock species + Mgt zone + Prevention + Farm residency + Season + Year + Season*Livestock species	0.779	8	−628.240	1297.3	1.57	0.180
Livestock species + Mgt zone + Prevention + Farm residency + Season + Year	0.638	7	−637.418	1297.8	2.02	0.143
**Model Coefficients**	**β**	**SE**	**95% Confidence Interval**			
			**Lower**		**Upper**	
(intercept)	7.537	17.643	−23.537		33.417	
Livestock species ^1^ (Sheep/goats)	0.551	0.175	0.181		0.911	
Livestock species (Horses)	−1.253	0.445	−2.205		−0.391	
Mgt zone (ZPE) ^2^	0.379	0.079	0.223		0.535	
Prevention (Yes) ^3^	−1.263	0.160	−1.577		−0.948	
Farm residency ^4^ (Transhumant)	−0.883	0.124	−1.125		−0.638	
Season ^5^ (Spring)	−0.076	0.296	−0.619		0.544	
Season (Early summer)	0.279	0.212	−0.167		0.686	
Season (Late summer)	0.703	0.182	0.321		1.062	
Season (Winter)	−1.782	0.713	−3.151		−0.297	
Year	−0.004	0.009	−0.035		0.0140	
Season (Spring)*Livestock species (Sheep/goats)	−0.516	0.519	−1.596		−0.201	
Season (Early summer)*Livestock species (Sheep/goats)	0.159	0.245	−0.251		0.806	
Season (Late summer)*Livestock species (Sheep/goats)	0.071	0.198	−0.366		0.614	
Season (Winter)*Livestock species (Sheep/goats)	−0.567	0.916	−3.003		1.026	
Season (Spring)*Livestock species (Horses)	0.216	0.574	−1.034		1.785	
Season (Early summer)*Livestock species (Horses)	0.206	0.487	−0.830		1.535	
Season (Late summer)*Livestock species (Horses)	0.391	0.521	−0.351		1.713	
Season (Winter)*Livestock species (Horses)	−6.414	355.68	−935.221		912.883	

^1^ Reference: Cattle; ^2^ Reference: Park; ^3^ Reference: No prevention; ^4^ Reference: Resident; ^5^ Reference: Autumn. *: interaction (multiplicative effect).

**Table A3:** Impact of Apennine bears on the livestock sector in the Abruzzo Lazio and Molise National Park (central Italy, 2005–2015), as determined by the compilation of 1057 verified records.

Year	Depredation Events (n)						Depredated Heads of Livestock (n)					Compensation Costs (€)					
	Total ^1^	Cattle	Horses	Sheep	Goats	Other	Cattle	Horses	Sheep	Goats	Other	Total ^2^	Cattle	Horses	Sheep	Goats	Other
2005	69	20	7	23	5	5	20	7	56	33	12	29,060	10,683	4345	5292	1951	1770
2006	88	37	9	30	2	2	38	9	71	9	3	32,087	14,864	4752	7754	260	790
2007	71	32	8	26	2	−	33	8	60	13	−	32,005	15,257	6499	7799	1040	−
2008	144	26	18	79	14	2	27	19	158	23	2	50,459	15,101	8660	21,788	2029	570
2009	75	11	3	54	1	−	12	3	107	11	−	26,565	4620	1086	16,679	156	−
2010	139	31	6	77	15	−	33	6	188	37	−	72,678	29,957	2722	28,144	3959	−
2011	155	62	10	67	12	2	70	10	122	17	2	91,891	57,717	5477	23,801	2901	900
2012	97	27	5	50	7	1	30	6	100	14	1	43,570	20,664	2761	12,828	1434	420
2013	105	25	14	41	20	−	29	16	84	47	−	48,741	16,001	7886	14,653	6816	−
2014	66	24	10	21	6	4	28	10	34	6	6	35,508	20,645	5324	6011	1250	1800
2015	48	27	4	13	2	−	32	4	25	6	2	34,865	24,843	3596	4491	585	−
*mean*	*96*	*29*	*9*	*44*	*8*	*1*	*32*	*9*	*91*	*20*	*3*	*45,221*	*20,941*	*4828*	*13,567*	*2035*	*1042*
*(±SD)*	*(34)*	*(12)*	*(4)*	*(22)*	*(6)*	*(2)*	*(14)*	*(5)*	*(48)*	*(13)*	*(3)*	*19,383*	*13,309*	*2173*	*7811*	*1860*	*547*

^1^ Including 58 cases that could not be classified at the species level. ^2^ Including 31,082 € ($\bar{x}$ = 3282 ± 2145 €/year) referred to cases that could not be classified at the species level.
